# Interaction of translationally controlled tumor protein with Apaf-1 is involved in the development of chemoresistance in HeLa cells

**DOI:** 10.1186/1471-2407-14-165

**Published:** 2014-03-07

**Authors:** Jaehoon Jung, Hyo Young Kim, Jeehye Maeng, Moonhee Kim, Dong Hae Shin, Kyunglim Lee

**Affiliations:** 1 Graduate School of Pharmaceutical Sciences, College of Pharmacy, Ewha Womans University, Seoul 120-750, Korea

**Keywords:** Apaf-1, Apoptosis, Cancer, Chemoresistance, TCTP

## Abstract

**Background:**

Translationally controlled tumor protein (TCTP), alternatively called fortilin, is believed to be involved in the development of the chemoresistance of tumor cells against anticancer drugs such as etoposide, taxol, and oxaliplatin, the underlying mechanisms of which still remain elusive.

**Methods:**

Cell death analysis of TCTP-overexpressing HeLa cells was performed following etoposide treatment to assess the mitochondria-dependent apoptosis. Apoptotic pathway was analyzed through measuring the cleavage of epidermal growth factor receptor (EGFR) and phospholipase C-γ (PLC-γ), caspase activation, mitochondrial membrane perturbation, and cytochrome c release by flow cytometry and western blotting. To clarify the role of TCTP in the inhibition of apoptosome, in vitro apoptosome reconstitution and immunoprecipitation was used. Pull-down assay and silver staining using the variants of Apaf-1 protein was applied to identify the domain that is responsible for its interaction with TCTP.

**Results:**

In the present study, we confirmed that adenoviral overexpression of TCTP protects HeLa cells from cell death induced by cytotoxic drugs such as taxol and etoposide. TCTP antagonized the mitochondria-dependent apoptotic pathway following etoposide treatment, including mitochondrial membrane damage and resultant cytochrome c release, activation of caspase-9, and -3, and eventually, the cleavage of EGFR and PLC-γ. More importantly, TCTP interacts with the caspase recruitment domain (CARD) of Apaf-1 and is incorporated into the heptameric Apaf-1 complex, and that C-terminal cleaved TCTP specifically associates with Apaf-1 of apoptosome in apoptosome-forming condition thereby inhibiting the amplification of caspase cascade.

**Conclusions:**

TCTP protects the cancer cells from etoposide-induced cell death by inhibiting the mitochondria-mediated apoptotic pathway. Interaction of TCTP with Apaf-1 in apoptosome is involved in the molecular mechanism of TCTP-induced chemoresistance. These findings suggest that TCTP may serve as a therapeutic target for chemoresistance in cancer treatment.

## Background

Translationally controlled tumor protein (TCTP), also called fortilin, P23, and histamine-releasing factor (HRF), is a housekeeping protein, highly conserved in humans to plants, has been shown to play pleiotropic functions in cell growth, proliferation, and apoptosis among others, in response to wide-ranging signals (reviewed in [1]). Our earlier work [2], has shown that post-translational modifications of TCTP such as proteolysis and dimerization are prerequisites that endow TCTP with its plethora of functions.

TCTP is significantly overexpressed in tumor cells while suppression of TCTP expression enhances apoptosis and causes reversion of transformed cells to their normal phenotype [3-5]. TCTP exhibits its anti-apoptotic functions [3] through mechanisms that stabilize the anti-apoptotic Bcl-2 family protein, MCL1 [6] and that antagonize the dimerization of pro-apoptotic Bax [7]. The N-terminal region of TCTP is known to be involved in the antiapoptotic mechanism via interaction with antiapoptotic Bcl-xL [8]. As a Ca^2+^-binding protein [9], TCTP sequesters the intracellular Ca^2+^ to perturb the Ca^2+^-dependent apoptosis [10]. In addition, recent reports suggest that TCTP represses the tumor suppressor p53 in a reciprocal mode [11,12]. We recently showed that Na,K-ATPase, an interacting partner in tumorigenesis, directly associates with TCTP to induce human breast epithelial cell transformation through Src-dependent EGFR transactivation [13].

Also, it has been reported that TCTP overexpression induces chemoresistance by protecting the various tumor cells against DNA-damaging agents such as etoposide or 5-fluorouracil and against tunicamycin-induced endoplasmic reticulum (ER) stress [3,14,15]. Of interest, increased expression of TCTP is revealed to have an interrelation with increased chemoresistance, of malignant melanoma to cytotoxic agents such as etoposide and its increased survival [16]. In addition, a temporal proteome profiling upon taxol exposure, revealed increased TCTP expression during apoptosis [17]. Studies of colon cancer cell response to oxaliplatin treatment revealed temporal upregulation of TCTP [18]. However, little is known about the unique molecular mechanisms in the role of TCTP in the development of the chemoresistance of tumor cells against anticancer drugs such as etoposide, taxol, and oxaliplatin.

In this context, the role of deregulation or defects of apoptosome function in the development of chemoresistance [19], needs consideration as most anticancer drugs suggested to mediate cell death via mitochondrial apoptotic pathway [20]. For example, inactivation or silencing of apoptotic protease activating factor (Apaf-1), has been implicated in the development of chemoresistance [21] by metastatic malignant melanomas [22]. Inhibition of Apaf-1 provides a preferential survival advantage to neoplastic cells [23] and the lack of cytosolic Apaf-1 due to its sequestration in lipid raft is noted as a new mechanism of chemoresistance in B lymphoma [24]. Acquired cisplatin resistance is partially reversed when Apaf-1 is exogenously overexpressed in HeLa cells [25]. Furthermore, it has been shown that apoptosome-dependent apoptosis can be inhibited when Apaf-1 is exposed to endogenous regulators including Bcl-xL [26], Heat shock protein 70 (HSP 70) [27], and Apaf-1 interacting protein (APIP) [28].

We hypothesize that TCTP may be another possible regulator of Apaf-1 that binds to Apaf-1 to incorporate into apoptosome complex. In the present study we tested this hypothesis by investigating whether TCTP involves in the development of chemoresistance in mitochondria-mediated apoptosis. We specifically examined the role of TCTP in apoptosome inhibition, by studying its structural modification in etoposide-treated cancer cells.

## Methods

### Reagents and antibodies

Antibody detecting anti-Na,K-ATPase α1 subunit was purchased from Upstate (Billerica, MA). Anti-PLC-γ, -actin, -His, -cytochrome c, -caspase-9, -cleaved caspase-3, -cleaved caspase-7, -cleaved caspase-9, -cleaved PARP, -Flag, and -Apaf-1 antibodies were from Cell Signaling Technology (Boston, MA). Anti-EGFR, and -GFP antibodies were from Santa Cruz (Santa Cruz, CA). Anti-TCTP-specific antibody was from LabFrontier (Korea). 5,5′,6,6′-tetrachloro-1,1′,3,3′-tetraethylbenzimidazolylcarbocyanine iodide (JC-1) was from Molecular Probe (Carlsbad, MO). Etoposide, Taxol (paclitaxel), Ac-LEHD and Ac-DEVD were from Calbiochem (San Diego, CA). Bovine serum albumin (BSA), dATP, cytochrome c, and carbonyl cyanide *m*-chlorophenylhydrazone (CCCP) were from Sigma (St Louis, MO). Anti-OxPhos Complex IV (COX IV) antibody was from Invitrogen (Carlsbad, CA). Purified WD Repeat (WDR) protein was from Abnova Corporation (Taiwan).

### Cell culture and infection

HeLa cells that were from American Type Culture Collection (ATCC) were maintained and cultured in a Dulbecco’s modified Eagle medium (DMEM) supplemented with 10% fetal bovine serum (FBS), penicillin (100 U/ml), and streptomycin (100 μg/ml). Cells were placed at 37°C in a 5% CO_2_ atmosphere incubator with humidification. For adenoviral expression, cells were infected with 10 multiplicity of infection (MOI) of adenoviruses containing N-terminal Flag-tagged TCTP or C-terminal GFP-tagged TCTP genes, or with its corresponding null virus for 2 h in DMEM without serum at 37°C in 5% CO_2_, followed by 20 h incubation in DMEM media containing 10% serum. The cells were then serum-starved for 2 h prior to drug treatment. The level of *TCTP* overexpression was determined by western blot analysis. Etoposide (20 μM) was administered after infection of adenovirus.

### Cell death analysis

For detection of apoptosis, HeLa cells were seeded onto 12-well plates and treated with etoposide (20 μM) or taxol (0.1 μM) for an indicated time. To measure the DNA fragmentation by apoptosis, cells were stained with propidium iodide (PI) and were assayed under fluorescence-activated cell sorting (FACS) analysis. Following the treatment with cytotoxic agents, HeLa cells were harvested, and reconstituted in ice-cold phosphate buffered saline (PBS) supplemented with 50 μg/ml of PI. Samples were then detected their fluorescence by flow cytometry (FACS Calibur, BD) and the results were analyzed using WinMDI software.

### Immunoprecipitation and western blotting

Under the presence of dATP and cytochrome c, HeLa S-100 extract was incubated with recombinant human TCTP for 1 h at 4°C in PBS. The reaction mixtures were subjected to preclearance by adding Protein G-agarose (Roche, IN) and incubated for 3 h at 4°C on a rocking platform, to remove the non-specific protein binding to agarose. After eliminating the Protein G beads by centrifugation at 14,000 × g for 10 min (4°C), anti-Apaf-1 antibody was incubated with Protein G-agarose for overnight, followed by the adding of HeLa S-100 extract into the reaction mixture for 1 h (4°C). The immune complexes resulted were pelleted, washed three times with ice-cold PBS, reconstituted with SDS sample buffer and then resolved on the SDS-PAGE. Western blotting of lysates from GFP-tagged TCTP-overexpressing cells following etoposide treatment was performed by anti-GFP- and protein-specific antibodies. Western blotting and immunoprecipitation of lysates from Flag-tagged adNull- and adTCTP-infected cells following etoposide treatment were performed by using anti-Na,K-ATPase α1, Apaf-1 and protein-specific antibodies. Image of western blot was visualized and obtained using LAS-3000 image analysis system (Fujifilm Life Science).

### In vitro activation of apoptosome formation

To obtain the S-100 extract, HeLa cells were harvested through centrifugation. After washing the cells, cells were then resuspended in buffer (1.5 mM MgCl_2_, 10 mM KCl, 20 mM HEPES (pH 7.5), 1 mM EGTA and EDTA, 0.1 mM phenylmethylsulfonyl fluoride (PMSF), 10 μg/ml leupeptin/aprotinin, and 1 mM dithiothreitol (DTT)). Then, reconstituted cells were homogenized with a Dounce glass homogenizer, and the resultant cell homogenates were subjected for centrifugation at 10,000 × g for 10 min (4°C) to extract the nuclear and mitochondrial organelles. The supernatants containing S-100 fraction were obtained and were mixed with 1 mM dATP/10 μM cytochrome c at a 2.5 mM Mg^2+^ concentration. Where indicated, recombinant TCTP protein was supplemented in the reaction mixture.

### Isolation of cytosolic and mitochondrial fractions

Following centrifugation, cells were harvested and the mitochondrial and cytosolic fractions were isolated using commercial kit (Pierce Biotechnology) according to the manufacturer’s instructions. In brief, cells were incubated with Reagent A for 2 min on ice and then transferred to Dounce homogenizer for homogenization (20 strokes). After adding the Reagent C, the mixtures were then centrifuged at 700 × g for 10 min at 4°C. The supernatant were then collected and further centrifuged at 3,000 × g for 15 min at 4°C to pellet the mitochondria. The resulting supernatant was designated as cytosolic fraction and the mitochondrial precipitate was washed with Reagent C followed by centrifugation at 12,000 × g for 5 min at 4°C. The purity of cytosolic and mitochondrial fractions was confirmed by the western blotting by detecting the immunoactivity of actin and COX IV, respectively.

### Measurement of cytochrome c release

Cytochrome c release was measured by western blotting or quantified using a fluorescent dye. Following the isolation of cytosolic and mitochondrial fractions from HeLa cells as described above, cytochrome c contents in each fraction were analyzed by immunoblot analysis using anti-cytochrome c-specific antibody (Cell Signaling Technology). To quantify the cytochrome c release, cells were mixed with a buffer containing 20 mM HEPES, 10 mM KCl, 1.5 mM MgCl_2_, 1 mM EGTA and EDTA, 1 mM AEBSF, 8 mM DTT, and 250 mM sucrose, supplemented with digitonin. Then the cells were harvested and subjected for fixation using 4% formaldehyde/1% fetal calf serum (FCS) solution. Permeabilized cells were incubated with 10% FCS in phosphate buffer and reacted with fluorescence-tagged anti-cytochrome c antibody. Cells were then washed and analyzed using flow cytometry (FACS Calibur, BD). Data obtained were presented in relative fluorescent units (RFUs).

### Analysis of mitochondrial membrane potential

To determine the perturbation of mitochondrial membrane potential (ΔΨ_m_), the fluorescent cationic dye, 5,5′,6,6′-tetrachloro-1,1′,3,3′-tetraethylbenzimidazolylcarbocyanine iodide (JC-1, Molecular Probes) was used with FACS. The red-to-green ratio of JC-1 fluorescence is detected to probe the ΔΨ_m_. When the integrity of mitochondrial membrane is maintained with high potential, JC-1 forms aggregates with red-fluorescence. But the perturbation of mitochondrial membrane leads it to emit only green fluorescence because of the loss of membrane potential. The mitochondrial membrane uncoupler, carbonyl cyanide *m*-chlorophenylhydrazone (CCCP), was used as a positive control that disturbs the mitochondrial membrane potential. After loading the HeLa cells with JC-1 at 37°C, cells were analyzed by FACS (FACS Calibur, BD) using FL-1 (JC-1 monomer, green) and FL-2 (JC-1 aggregate, red) channels.

### Identification of TCTP binding domain on Apaf-1

Several Apaf-1 deletion mutants that contain C-terminal His-tags were designed and constructed by referencing the previous study [29]. Recombinant proteins including Full APAF-1, APAF-530, APAF-420, and APAF-97 were expressed in BL21(DE3)pLysS *Escherichia coli* and purified through affinity purification on a Ni Sepharose beads. WD Repeat (WDR) of Apaf-1 protein was obtained from Abnova Corporation. GST-tagged recombinant TCTP protein was bacterially expressed in *E.coli* system and purified using GST fusion protein purification kit (Thermo Scientific). Purified His-Apaf-1 variants or WDR were immobilized on Handee™ spin column (PIERCE, IL) and then incubated with TCTP-GST protein. Each eluates were separated by SDS-PAGE and then subjected for silver staining.

## Results

### TCTP inhibits drug-induced cell death by inhibiting the fragmentation of EGFR and PLC-γ

**Figure 1:**
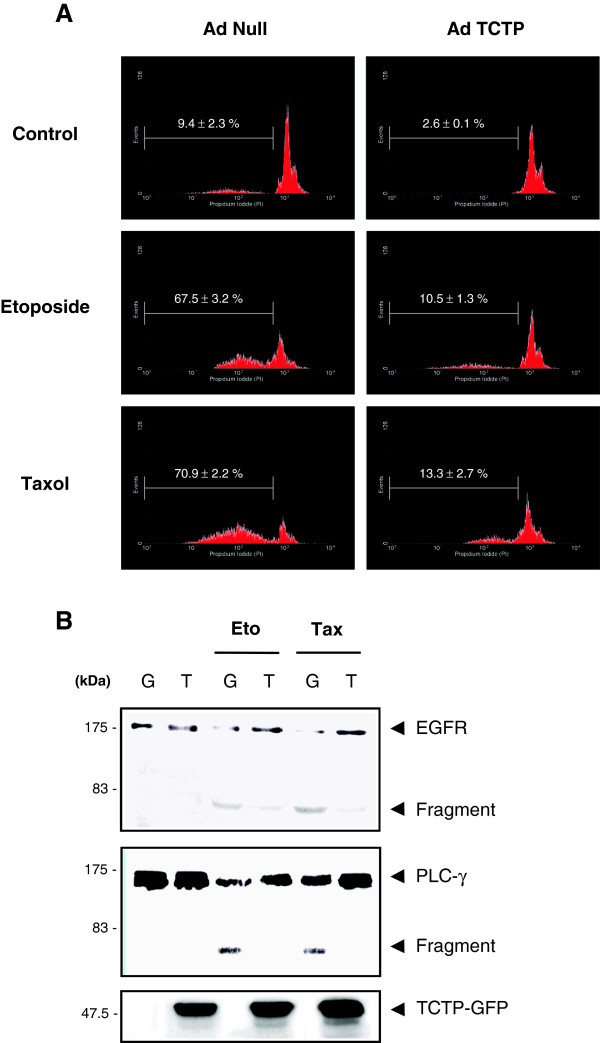
**TCTP inhibits anticancer drug-induced cell death and cleavage of EGFR and PLC-γ in HeLa cells. (A)** TCTP-induced inhibition of cytotoxic drug-induced cell death. AdNull- and adTCTP-infected HeLa cells (MOI, 10) were incubated with 20 μM etoposide or 0.1 μM taxol and then DNA fragmentation was analyzed using PI staining and FACS as described in Materials and Methods. **(B)** TCTP-induced inhibition of cytotoxic drug-induced EGFR and PLC-γ fragmentation. After treatment of 20 μM etoposide or 0.1 μM taxol, adGFP (G)- and adTCTP-GFP (T)-infected HeLa cell (MOI, 10) extracts were blotted with anti-EGFR, -PLC- γ, and -GFP antibodies.

We attempted to clarify the antiapoptotic role of TCTP in the development of chemoresistance to etoposide, an inhibitor of topoisomerase II, as well as taxol (paclitaxel), a microtubule stabilizer, those are widely used anticancer drugs with distinctive modes of action. Human *TCTP* gene was overexpressed in human cervical cancer cells (HeLa) using adenoviral infection and apoptosis was measured by DNA fragmentation using propidium iodide (PI) staining and fluorescence-activated cell sorting (FACS). We found that treatment with both etoposide and taxol increased cell death in HeLa cells. When TCTP was overexpressed, cell death decreased from 68% to 11%, in etoposide-treated HeLa cells and from 71% to 13%, in taxol-treated HeLa cells (Figure 1A), confirming that TCTP inhibits cytotoxicity and cell death induced by two anticancer drugs.

It has been suggested that etoposide as well as taxol cause apoptosis via caspase activation [30,31]. Because EGFR and PLC-γ are known to be cleaved by caspases during apoptotic process [32,33], we examined whether TCTP also inhibits the fragmentation of these proteins. Figure 1B shows that treatment of cells with etoposide resulted in the fragmentation of both EGFR and PLC-γ and that overexpression of TCTP decreased such fragmentations. Taken together, these findings suggest that TCTP enabled the HeLa cells to acquire chemoresistance in etoposide-induced apoptosis possibly through inhibition of initiator or effector caspase activity thereby preserving the key players for tumor cell function such as EGFR and PLC-γ.

### TCTP inhibits mitochondrial membrane perturbation thereby reducing cytochrome c release from mitochondria to cytosol in etoposide-induced cell death

**Figure 2:**
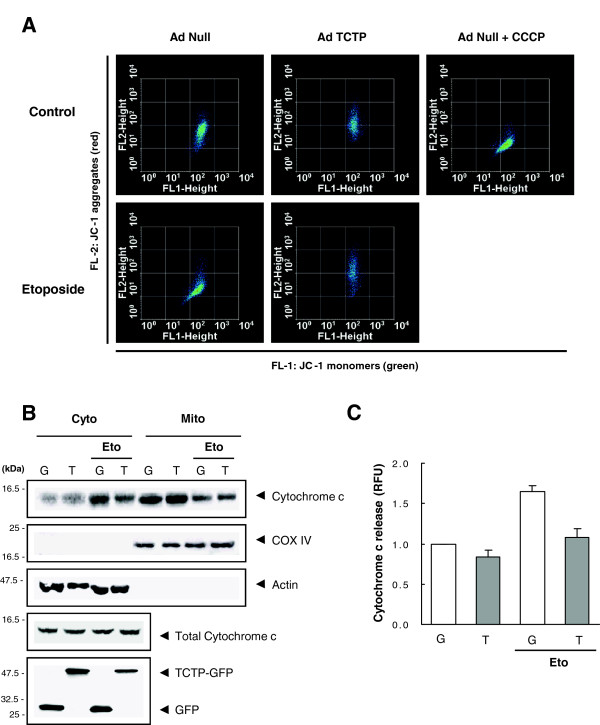
**TCTP inhibits mitochondrial cytochrome c release and mitochondrial membrane depolarization in etoposide-induced cell death. (A)** TCTP-induced inhibition of mitochondrial membrane perturbation. AdNull and adTCTP-infected cells (MOI, 10) were treated with 20 μM etoposide. Loss of mitochondrial membrane potential was measured using JC-1 dye by flow cytometry. The disrupter, CCCP was used as a positive control. JC-1 forms aggregates in the high mitochondrial membrane potential whereas the disrupted potential during apoptosis leads to form the JC-1 monomer. Loss of membrane potential was detected by measuring the shift of fluorescence from FL-2 (JC-1 aggregates, red fluorescence) to FL-1 (JC-1 monomers, green fluorescence) in FACS analysis. **(B)** TCTP-induced inhibition of mitochondrial cytochrome c release. AdGFP (G)- and adTCTP-GFP (T)-infected cells (MOI, 10) were treated with 20 μM etoposide. After fractionation of mitochondrial and cytosolic fractions, anti-cytochrome c antibodies were used for detecting its contents by western blot analysis. **(C)** Cytochrome c release was quantitated by expressing as RFU. AdGFP (G)- and adTCTP-GFP (T)-infected cells (MOI, 10) were treated with 20 μM etoposide. Following incubation with fluorescence-tagged cytochrome c-specific antibody, cells were subjected to FACS analysis. Data represent cytochrome c release relative to the control (mean ± S.D.) of two independent experiments.

Following Bax translocation to mitochondria, release of intermembrane cytochrome c lead to the perturbation of the mitochondrial membrane potential by disturbing the electron transfer [34]. We tested whether TCTP inhibits mitochondrial membrane polarization during etoposide-induced cell death. Flow cytometry, employing the dye, JC-1, an indicator for mitochondrial membrane potential, showed that the distribution of membrane potential is normal in untreated HeLa cells (Figure 2A). In contrast, the distribution of membrane potential shifted from red (FL-2) to green (FL-1) fluorescence when carbonyl cyanide *m*-chlorophenylhydrazone (CCCP), a protonophore, was treated as a positive control, through its effects on mitochondrial uncoupling (Figure 2A). Interestingly, hyperpolarized distribution of mitochondrial membrane potential in etoposide-induced cell death was inhibited by TCTP overexpression (Figure 2A).

Next, we examined if TCTP also inhibits cytochrome c release under genotoxic stress. As shown in Figure 2B, the content of cytochrome c was higher in the cytosolic fraction of etoposide-treated HeLa cells than in untreated cells. In contrast, TCTP overexpression inhibited the cytochrome c release from mitochondria to cytosolic fraction in etoposide-treated HeLa cells (Figure 2B), as also confirmed by the fluorimetry analysis using fluorescence-tagged anti-cytochrome c antibody (Figure 2C). Therefore, TCTP appears to induce the chemoresistance of etoposide-induced cell death by inhibiting the mitochondrial membrane damage (Figure 2A) and the resultant cytochrome release into cytosolic fraction (Figure 2B and C).

### TCTP inhibits caspase activation in etoposide-induced cell death

**Figure 3:**
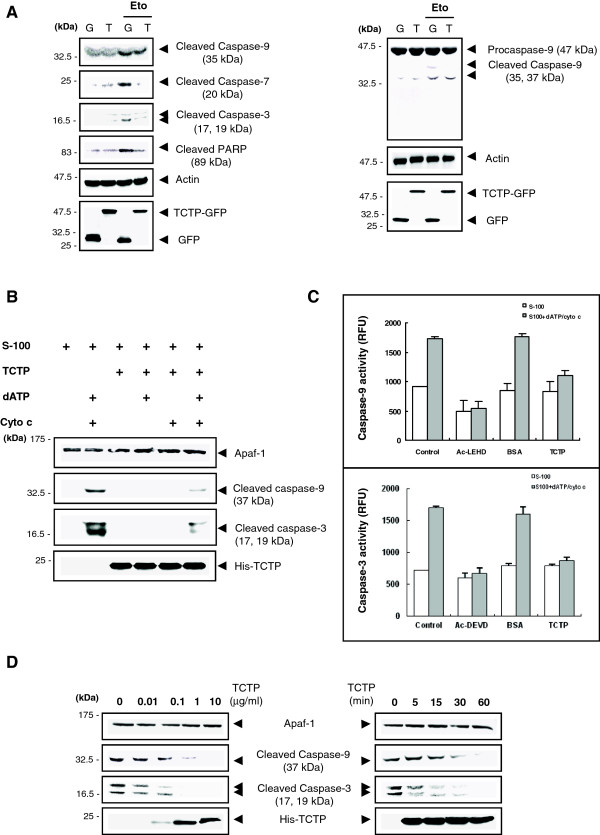
**TCTP inhibits caspase activation in etoposide-induced cell death. (A)** TCTP-induced inhibition of caspase-9, -7, and -3 fragmentation. AdGFP (G)- and adTCTP-GFP (T)-infected cells (MOI, 10) were treated with 20 μM etoposide and the activation of caspases were determined by western blot assay using cleaved form-specific caspase-9, -7, and -3, and PARP antibodies (*left panel*). Cleavage of procaspase-9 was further assayed using specific antibodies detecting 35- and 37-kDa form of cleaved caspase-9 (*right panel*). **(B)** TCTP-induced inhibition of caspase-9 and -3 cleavages under in vitro assays of apoptosome reconstitution. S-100 extracts were incubated with dATP/cyto c to induce the apoptosome formation. Activation of caspase-9 and -3 was detected by western blotting in the presence or absence of TCTP in the reaction. **(C)** TCTP-induced inhibition of caspase-9 and -3 activities confirmed by inhibitor assay. The specific inhibitor of caspase-9 and -3, Ac-LEHD and Ac-DEVD, respectively, were used. Caspase activity was presented as RFU by using fluorogenic substrates for caspases. Error bar represent SD of two independent experiments. **(D)** Time- and dose-dependence of TCTP-induced inhibition of caspase-9 and -3 fragmentations. Following dose- and time-dependent incubation with His-tagged TCTP at a time of 30 min and at a dose of 1 μg/ml, respectively, western blot assay was performed to detect caspase activity using anti-Apaf-1, -cleaved caspase-9, -cleaved caspase-3, and -His-specific antibodies.

Release of cytochrome c from mitochondria, induces the formation of functional apoptosome that signals the activation of caspase cascade in the mitochondria-dependent apoptotic pathway [35,36]. Apoptosome cleaves apical caspase-9, which in turn induces the activation of caspases -3 and -7 to execute the dismantling of the cells (reviewed in [37]) through proteolysis of its target proteins such as poly ADP ribose polymerase (PARP) [38]. In etoposide-treated human melanoma cells, cytochrome c release was observed along with upregulation of caspases -9 and -3 [39]. Because TCTP inhibits cytochrome c release from the mitochondria, effects on caspase activity by TCTP were investigated by specifically detecting the cleaved form of caspases. As presented in Figure 3A, etoposide treatment induced cleavage of caspase -9, -3, and -7 as well as fragmentation of its target PARP in HeLa cells. Adenoviral overexpression of TCTP inhibited the production of all of these fragments except for the 35 kDa of caspase-9 (Figure 3A, *right panel*) in etoposide-treated cells.

Reconstitution of the caspase activation in vitro was performed to confirm the inhibitory effect of TCTP on the caspase activity. In the presence of cytochrome c and dATP, Apaf-1 oligomerizes to assembly into a heptameric apoptosome complex [35]. Cytosolic environment of apoptosome assembly was artificially reconstituted by using S-100 extracts that is mitochondria-depleted cytosolic fraction of HeLa cells. Only when both dATP and cytochrome c were added into S-100 cytosolic extract, Apaf-1 monomer in S-100 extract formed an apoptosome in vitro thereby producing the cleaved form of caspase-9 and caspase-3 (Figure 3B). Consistent with the result in Figure 3A, preincubation of recombinant TCTP with the reaction mixture attenuated the activation of caspase-9 as well as caspase-3 (Figure 3B). To note, cleaved form of TCTP was detected when S-100 was incubated with TCTP (Figure 3B and Additional file 1: Figure S1).

To ascertain the effect of TCTP on caspase activity, caspase-specific inhibitors were added into the reactions and caspase activity was determined using a fluorogenic substrate that emits fluorescence when caspases cleave it. Caspase-9-specific inhibitor, Ac-LEHD, in S-100 with dATP/cytochrome c decreased the caspase-9 activity to an extent comparable to that of S-100 control (Figure 3C, *upper panel*). Addition of TCTP protein reduced the caspase-9 activity specifically in apoptosome-forming condition, while BSA, a protein control, had a minimal effect compared to that of control (Figure 3C, *upper panel*). TCTP also inhibited caspase-3 in apoptosome-containing cells to the similar extent as caspase-3 inhibitor, Ac-DEVD-treated cells (Figure 3C, *lower panel*). As shown in Figure 3D, TCTP inhibited caspase-9, and -3 fragmentations in a dose- and a time-dependent manner.

### TCTP interacts with caspase recruitment domain (CARD) of Apaf-1 in the apoptosome complex to inhibit the activation of caspase-9

**Figure 4Figure 4:**
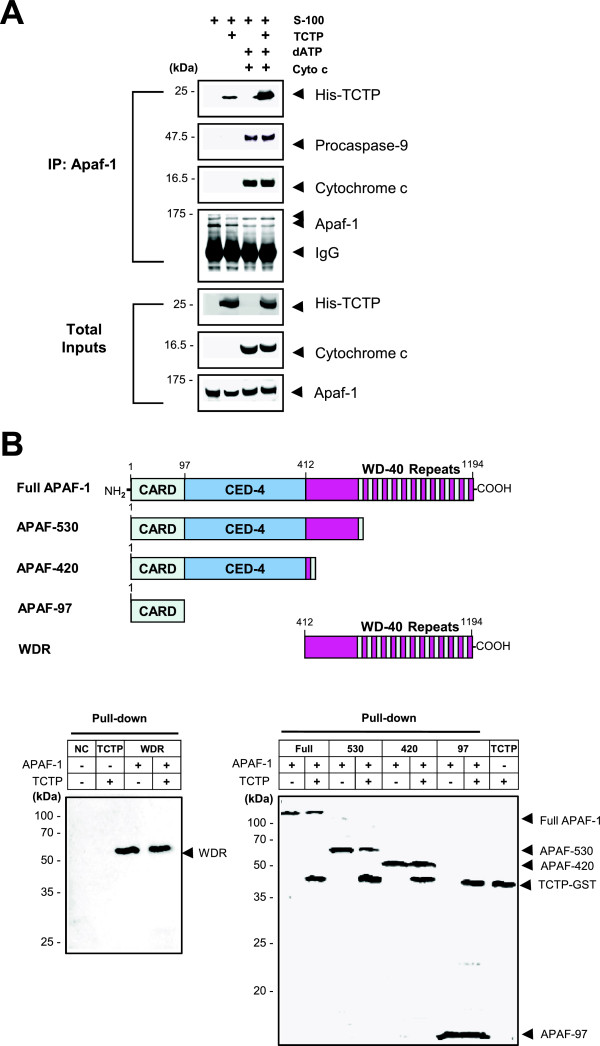
**TCTP interacts with CARD of Apaf-1 to form an apoptosome complex. (A)** Binding of TCTP to Apaf-1 in apoptosome-forming condition. TCTP, procaspase-9, and cytochrome c binding to Apaf-1 was analyzed. Recombinant human TCTP, cytochrome c, dATP and S-100 extract were incubated, immunoprecipitated with anti-Apaf-1-specific antibody and then blotted with antibodies, as described in Methods. **(B)** Interaction of TCTP with CARD of Apaf-1. Schematic diagram of full-length Apaf-1 and its variants devoid of particular domain(s) was presented. The full-length Apaf-1 (residues 1-1194) contains CARD (residues 1-97), CED-4 (residues 98-412), and WD-40 repeats (residues 413-1194). Recombinant Full APAF-1, APAF-530 (residues 1-530), APAF-420 (residues 1-420), APAF-97 (residues 1-97), and TCTP-GST (GST-tagged TCTP) were constructed. Purified WDR (WD Repeat) protein lacking both CARD and CED-4 was obtained from Abnova Corporation. Binding of TCTP-GST and His-tagged Apaf-1 constructs was analyzed using pull-down assay. Protein binding complexes were separated on SDS-PAGE and stained with silver. Purified TCTP-GST protein was also resolved by SDS-PAGE for detection of TCTP (*last lane*). NC, negative control.

TCTP may exert its antiapoptotic activity by inhibiting the caspase-9 activation in apoptosome, following etoposide treatment. To examine the mechanism of apoptosome inhibition by TCTP, we investigated whether TCTP interacts with Apaf-1 in vitro. S-100 extracts were incubated with dATP and cytochrome c to assemble the apoptosome, following pre-incubation with recombinant TCTP protein. The resulting protein complex was immunoprecipitated with anti-Apaf-1-specific antibodies. We found that Apaf-1 in S-100 extracts was bound to procaspase-9, cytochrome c and addition of TCTP to the mixture did not affect the binding of procaspase-9 and cytochrome c to the Apaf-1 in vitro, suggesting that TCTP did not inhibit the procaspase-9 binding to Apaf-1 (Figure 4A).

In order to identify which domain of Apaf-1 serves as the binding site for TCTP, we generated variants of full-length Apaf-1 devoid of some particular domain(s) present in Apaf-1, for example APAF-530 (residues 1-530), APAF-420 (residues 1-420), and APF-97 (residues 1-97) (Figure 4B), as previously described [29]. Recombinant full APAF-1 (residues 1-1194), APAF-530 (residues 1-530), APAF-420 (residues 1-420), APF-97 (residues 1-97), and TCTP-GST were expressed in *Escherichia coli*, and subjected to affinity purification (Additional file 2: Figure S2). His-tagged Apaf-1 variants were immobilized on a spin column and then incubated with or without GST-tagged TCTP. Silver staining of the eluates revealed that TCTP interacts with all of Apaf-1 variants, suggesting the interaction of TCTP at the site of Apaf-1 CARD (Figure 4B). A parallel experiment using WD Repeat (WDR), protein lacking CARD and CED-4 domains of Apaf-1, confirmed that CARD domain serves the site for TCTP binding to Apaf-1 (Figure 4B). Therefore, it appears that TCTP itself interacts with CARD of Apaf-1 to assemble into the apoptosome without interrupting the procaspase-9 binding to Apaf-1 in apoptosome-forming condition.

### Fragmented TCTP specifically interacts with Apaf-1 in etoposide-induced cell death

**Figure 5:**
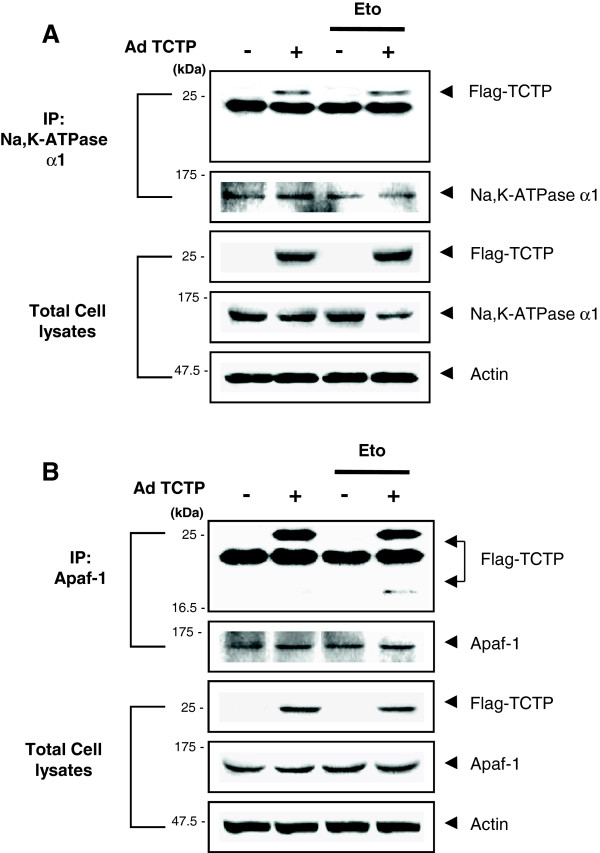
**Cleaved TCTP specifically binds to Apaf-1 during apoptosis. (A)** Full-length TCTP binding to Na,K-ATPase α1 subunit in TCTP-overexpressing HeLa cells in etoposide-induced cell death. Following treatment with 20 μM etoposide, Flag-tagged adNull- and adTCTP-infected cell extracts (MOI, 10) were immunoprecipitated with anti-Na,K-ATPase α1-specific antibodies and blotted with anti-Flag-specific antibody. **(B)** Fragmented TCTP binding to Apaf-1 in etoposide-induced cell death. Following treatment with 20 μM etoposide, Flag-tagged adNull- and adTCTP-infected cell (MOI, 10) extracts were immunoprecipitated with anti-Apaf-1-specific antibody and blotted with anti-Flag antibody.

Since fragmented form of TCTP was a component of in vitro reconstituted apoptosome complex (Figure 3B), cleaved TCTP may presumably operate in association with Apaf-1 in response to apoptotic trigger while full-length TCTP interacts with Na,K-ATPase [40]. To differentiate the interaction between Na,K-ATPase and TCTP upon etoposide treatment, we performed immunoprecipitation using anti-Na,K-ATPase antibodies following the adenoviral infection of N-terminal Flag-tagged TCTP. As shown in Figure 5A, Na,K-ATPase interacted with full-length TCTP and etoposide treatment had no effect on this binding. When the Apaf-1-interacting molecules were precipitated in parallel experimental settings, full-length TCTP found to be associated with Apaf-1 in the TCTP-overexpressing untreated cells. Treatment with etoposide resulted in additional interaction of Apaf-1 with short-length Flag-TCTP (Figure 5B).

Taken together these findings suggest that full-length TCTP binds to Apaf-1 CARD both in normal and apoptotic conditions whereas C-terminal cleaved TCTP (the Flag-tagging is located in the N-terminal of TCTP) specifically binds to Apaf-1 under apoptosome-forming conditions in TCTP overexpressed cells. It can be inferred that when apoptotic signaling is introduced, cleaved form of TCTP binds to Apaf-1 of apoptosome to interfere with the activation of mitochondria-mediated cell death, while full-length TCTP is responsible for Na,K-ATPase binding.

## Discussion

Genotoxic stress or DNA damage resulting from chemotherapy activates the intrinsic apoptotic pathway which includes a sequential cascade of events leading to cell death. Defects in the apoptotic pathways have been associated with tumorigenesis as well as resistance against conventional chemotherapeutics [19]. Downregulation of target enzyme topoisomerase II, modulation of microRNA, and acquisition of multiple drug resistance (MDR) phenotype through induction of *mdr-1* and ABC transporter genes [41-45] are the major known mechanism of resistance to etoposide treatment in tumor cells. In the present study, TCTP protected cancer cells from etoposide-induced cytotoxicity via sequential regulation of major events of mitochondrial apoptosis. TCTP overexpression (a) reduced mitochondrial membrane damage thereby preventing cytochrome c release into the cytosol; (b) inhibited apoptosome functions including caspase-9 activation, which in turn inhibited caspase-3; (c) perturbed the cleavage of the proteolytic targets including EGFR and PLC-γ; and (d) eventually inhibited cell death in etoposide-treated HeLa cells.

Most importantly, TCTP seems to inhibit the etoposide-induced cell death at the site of apoptosome formation via association with Apaf-1. Though previous studies indicated that abnormal function of Apaf-1 correlates with loss of sensitivity upon cytotoxic therapy [46] and that upregulation of TCTP is also related to the pathogenesis of chemoresistance in cancer cells [16], the exact molecular mechanisms and interactions are unknown. One example of negative regulation of apoptosome formation is the finding that constitutive overexpression of HSP70 is related to the resistance to apoptosis exhibited by particular tumor cells. HSP70 in part modulates the Apaf-1 function through its direct association with the CARD of Apaf-1, thereby inhibiting the oligomerization of Apaf-1 and association of Apaf-1 with procaspase-9 [47].

To clarify the interaction of TCTP with Apaf-1, studies are underway to test whether endogenous TCTP also binds to Apaf-1 in HeLa cells. Because we observed Apaf-1 binding and truncation of TCTP in a single cell line following TCTP overexpression, other types of etoposide-resistant cancer cells need to be explored for verifying the Apaf-1-TCTP interaction. Perturbation of apoptosis by Apaf-1-TCTP complex possibly involves the direct mechanism that inhibits the apoptosome. In addition, indirect mechanisms involving the regulation of certain molecules in apoptotic pathway may constitute the TCTP-induced chemoresistance. As HeLa cells are reported to retain the p53-mediated pathway [48,49], inhibition of p53 by TCTP is a possible mechanism that contributes to the TCTP-induced chemoresistance through the regulation of MDM2 or Bax. To clarify this, our future work will include the study of TCTP-Apaf-1 interaction using the p53-silenced cells.

Though TCTP associates with CARD region of Apaf-1 (Figure 4B) to which caspase-9 binds, this interaction did not inhibit the caspase-9 binding to Apaf-1 (Figure 4A) but abolished the production of active caspases (Figure 3B). Following etoposide treatment, procaspase-9 was cleaved to 35 and 37 kDa fragments (p35 and p37, respectively) but TCTP did not inhibit the production of p35 (Figure 3A, *right panel*). Initially, interaction of procaspase-9 with Apaf-1 induces auto-cleavage at Asp-315 of procaspase-9 to produce the p35. Once caspase-3 is activated by p35-containing apoptosome, it mediates another cleavage of caspase-9 at Asp-330, which produces p37 to amplify the apoptotic signal via positive feedback on the remaining procaspase-9 [29,35,36,50]. Therefore, selective inhibition of TCTP on the cleavage of procaspase-9 into p37 in etoposide-treated cells indicates that TCTP may not be involved in the initial auto-catalysis but inhibits the amplification of caspase cascade as it interrupts the cleavage at Asp-330. This observation is consistent with the finding that TCTP did not inhibit the initial procaspase-9 interaction with Apaf-1 (Figure 4A). Taken together, it can be postulated that incorporation of cleaved TCTP into apoptosome inhibits etoposide-induced apoptosis by preventing recruitment of procaspase-3 to the apoptosome. In addition, the mechanism how fragmented TCTP that comprises only a small fraction of Apaf-1-binding TCTP in apoptosome enables the protection of HeLa cells against etoposide-induced toxicity remains to be addressed.

It can be inferred that some specific environment might led to posttranslational modification of TCTP possibly at its C-terminal. This possibility is supported by prior observations that N-terminal region of TCTP is necessary for its interaction with Bcl-xL for its antiapoptotic activity [8] and that extracellular N-terminal truncated TCTP exhibits cytokine-like activities via its homo-dimerization [2]. Therefore, cleaved form TCTP might contain N-terminal region and C-terminal fragments that possibly induce changes of ternary structure of TCTP which enable the association of Apaf-1 in apoptosome complex. This implies that distinctive form of TCTP may exist to separately interact with its different intracellular partners. The structural modification of TCTP including truncation or oligomerization, which may be provoked in specific conditions, appears to be involved in the mechanism for acquiring its pleiotropic activity.

Proteolytic enzymes, such as caspases, which are preferentially activated upon apoptotic signals, are possibly involved in the C-terminal cleavage of TCTP. However, this possibility can be ruled out since the primary sequence of TCTP does not contain the enzymatic site for caspase activity (data not shown). As only S-100 is competent to cleave the TCTP (Additional file 1: Figure S1), apoptosome formation is not necessary for its cleavage. We tested various kinds of protease inhibitors including VAD (a pan-caspase inhibitor), LDESD (a caspase-2/3 inhibitor), DEVD (a caspase-3 inhibitor), LEHD (a caspase-9 inhibitor), and MG132 (an inhibitor for calpain/proteasome) and found that cleavage of TCTP was not prevented by those agents. It is also supported by the evidence that recombinant caspase-3 did not cleave recombinant TCTP in vitro whereas it produces the cleavage of known substrate PARP [51]. Furthermore, S-100 treated with Apaf-1 domain-specific blocking antibodies as well Ca^2+^, followed by TCTP addition, caused the fragmentation of TCTP (data not shown). How TCTP becomes fragmented and which residues in TCTP are subjected to cleavage are under investigation.

Because TCTP is regarded as an oncogene and is up-regulated in tumors, reducing the TCTP levels or inhibiting its activity, are viewed as alternative rational approaches in cancer therapy. Recently, the pathophysiological association of high-TCTP status with poor prognosis in a large cohort of breast cancer patients was demonstrated [11]. Also, TCTP inactivation by pharmacological compounds has been studied as modalities of cancer therapy. For example, antidepressant sertraline and neuroleptic thioridazine, both of which restore p53, directly bind to TCTP to suppress TCTP-mediated activation of the p53 ubiquitination [11] and reduce the intracellular contents of TCTP, thus promoting apoptosis in tumor cells [52]. TCTP-binding antimalarial drug, artemisinin derivatives are being investigated as antitumor agents that have been tried as therapeutics in patients [53,54].

Most known functions of TCTP are related to its specific association with a variety of interacting molecules. The present study identified TCTP as a hitherto unknown partner of Apaf-1, and that it has a special role in the apoptosis induced by anticancer agents. It is logical to propose TCTP as a potential target of chemoresistance therapy because TCTP is shown to be incorporated into apoptosome complex to inhibit the etoposide-induced cell death. Therefore, the emerging picture from results described herein harnesses TCTP as potentially important clinical and pharmacological target not only in tumorigenesis but also in refractory cancers against chemotherapeutics.

## Conclusions

The current study indicates that TCTP is involved in the inhibition of etoposide-induced mitochondrial apoptosis in HeLa cells by perturbing the major events of the apoptotic pathway. In addition, TCTP is shown to interact with Apaf-1 CARD and is incorporated into the apoptosome complex in the apoptosome-forming conditions, thereby inhibiting the amplification of caspase cascade. Therefore, modulation of TCTP can be suggested as a potential strategy in the development of drugs to treat the tumorigenesis as well as chemoresistance.

## Abbreviations

Apaf-1: Apoptotic protease activating factor-1; APIP: Apaf-1 interacting protein; CARD: Caspase recruitment domain; EGFR: Epidermal growth factor receptor; HRF: Histamine-releasing factor; HSP 70: Heat shock protein 70; JNK: c-Jun NH_2_-terminal kinase; PARP: Poly ADP ribose polymerase; PLC-γ: Phospholipase C-γ; TCTP: Translationally controlled tumor protein.

## Competing interests

The authors have declared that no competing interest exists.

## Authors’ contributions

JJ and KL contributed in the design of the experiments and wrote the paper. JJ, HYK, and MK conducted experiments. JM and DHS contributed in the analysis of the results and prepared the paper. All authors read and approved the final manuscript.

## Pre-publication history

The pre-publication history for this paper can be accessed here:

http://www.biomedcentral.com/1471-2407/14/165/prepub

## Supplementary Material

Additional file 1: Figure S1 Cleavage of TCTP in S-100 extracts. To elucidate the necessary requirements for cleavage of TCTP, recombinant TCTP protein was incubated with various compositions of HeLa S-100 extracts, dATP, and cytochrome c. After incubation, the reaction mixtures were immunoblotted with anti-His-specific antibody. Click here for file

Additional file 2: Figure S2 Preparation of TCTP, Apaf-1 and its variants. TCTP, Apaf-1 and its variants were expressed in *Escherichia coli* system. Proteins were purified using its His- or GST-tagging on an affinity purification column, separated by 10% SDS-PAGE, and analyzed using the Silver Stain Plus kit. Click here for file
