# Supplementary figures and images for: Interaction of translationally controlled tumor protein with Apaf-1 is involved in the development of chemoresistance in HeLa cells
Source: BMC Cancer. 2014 Mar 7;14:165. doi: 10.1186/1471-2407-14-165 (PMC4015309; doi:10.1186/1471-2407-14-165)

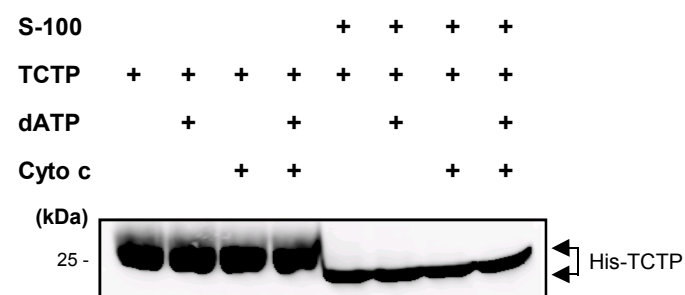

Supplement: Additional file 1: Figure S1 — Cleavage of TCTP in S-100 extracts. To elucidate the necessary requirements for cleavage of TCTP, recombinant TCTP protein was incubated with various compositions of HeLa S-100 extracts, dATP, and cytochrome c. After incubation, the reaction mixtures were immunoblotted with anti-His-specific antibody. [file 1471-2407-14-165-S1.pdf]

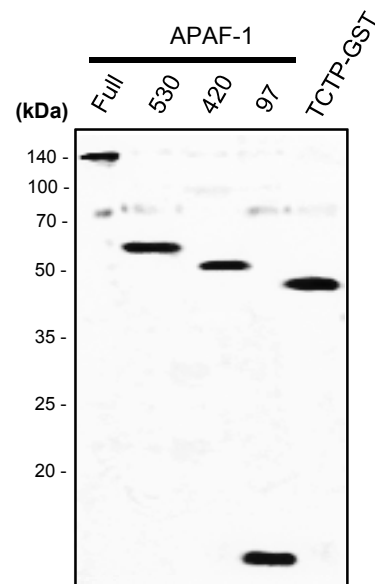

Supplement: Additional file 2: Figure S2 — Preparation of TCTP, Apaf-1 and its variants. TCTP, Apaf-1 and its variants were expressed in Escherichia coli system. Proteins were purified using its His- or GST-tagging on an affinity purification column, separated by 10% SDS-PAGE, and analyzed using the Silver Stain Plus kit. [file 1471-2407-14-165-S2.pdf]
